# Er:YAG Laser Therapy for Xanthelasma Palpebrarum: A Retrospective Case Series Evaluating Clinical Efficacy, Safety, and Patient Outcomes in Periocular Lesion Management

**DOI:** 10.1155/crdm/4616721

**Published:** 2025-06-09

**Authors:** Saman Al-Zahawi, Sara Masoomi, Ifa Etesami

**Affiliations:** Department of Dermatology/Razi Hospital, Tehran University of Medical Sciences (TUMS), Tehran, Iran

**Keywords:** Er:YAG laser, laser therapy, trichloroacetic acid, xanthelasma, xanthelasma palpebrarum

## Abstract

Xanthelasma palpebrarum (XP) is a benign condition of the eyelid skin, characterized by the appearance of well-defined, bilateral, yellow-colored papules or plaques in the periorbital area. Although patients often seek dermatological consultation for cosmetic reasons, the associated medical implications, especially in younger individuals, hold significant medical importance. XP can be treated through various methods such as surgical removal, trichloroacetic acid (TCA) 70% application, cryotherapy, and laser procedures. Among the laser therapies (CO_2_, pulsed dye laser, and Er:YAG), which have proven effective due to their convenient application and fewer complications, the Er:YAG laser stands out for its potential to deliver faster recovery, fewer pigmentation changes, minimal scarring, and a shorter period of postlaser redness. This study evaluated Er:YAG laser for XP in four cases. Three laser sessions effectively treated the lesions, leading to patient satisfaction despite incomplete clearance, with no recurrence in 12 months and no side effects. The study concluded that Er:YAG laser is preferred over other lasers for this condition due to its superior healing profile.

## 1. Introduction

XP is a benign eyelid skin condition characterized by well-defined, bilateral, yellow-colored papules or plaques in the periorbital area. Histologically, there is perivascular, periadnexal, superficial, and deep dermal fat deposition.

This deposition occurs mainly on the inner canthus of the eyes in middle-aged and elderly individuals. However, patients as young as 9 years have been reported [1]. The cosmetic concern of XP is the main reason behind consultation with dermatologists. Importantly, the associated medical conditions are of significant medical value, especially in younger individuals. To exclude such associations, it is highly recommended that patients with XP younger than 40 years undergo evaluation for inherited diseases of lipid metabolism (primary hyperlipidemia) and cardiovascular diseases [2]. Moreover, XP may be seen in the background of a normal lipid profile in nearly a quarter of patients [3].

An important entity to be distinguished from XP is the necrobiotic xanthogranuloma, which has a periorbital distribution pattern and clinical presentation such as XP, but importantly, it is associated with monoclonal gammopathy and other hematological malignancies [4].

Despite its benign and asymptomatic nature, the noticeable cosmetic changes of XP often prompt patients to seek early medical advice. Therefore, a comprehensive approach should first address any potential underlying systemic associations before selecting from the various available treatment options.

It is well known that when XP develops, lifestyle modification and lipid-lowering agents will not lead to clearance of the lesions, as XP lesions usually remain stable or may increase in size over time [5]. XP is treated by multiple modalities such as surgical excision, TCA, cryotherapy, and laser therapy. Among these modalities, the CO_2_ and Er:YAG lasers have emerged as effective modalities with substantial improvement and few side effects. Studies regarding the efficacy and safety of CO_2_ are numerous, while few studies have revealed the effects and safety of Er:YAG.

Here, we report four cases of XP with normal lipid profiles that responded well to Er:YAG laser therapy.

## 2. Case Presentation

Four female patients with no known medical condition, with a mean age of 35 years (range 28–42) and bilateral eyelid xanthelasma, were enrolled in the study. All patients gave informed consent. None of the patients were hyperlipidemic, neither had gammopathies, and none of them received treatment for XP. Patients were treated monthly with Er:YAG laser for 3 months. The 2940-nm Er:YAG laser (Fotona Dynamis ablative mode) with a 2-mm spot size, pulse duration SP mode (300 μs), and fluence 7 mJ/cm^2^ was applied. Based on our clinical experience with Fitzpatrick skin Type IV in this geographic region, the selected laser parameters demonstrated optimal safety and efficacy for the enrolled patients. Patients were advised to keep their eyes closed during the entire procedure, and eye shield protection was applied. Before the procedure, the lesions were marked with a surgical pen, and eutectic mixture of local anesthetics cream (EMLA) was applied 15 min before the procedure. The yellowish fatty tissue was carefully removed until pinpoint bleeding became visible, marking the endpoint of therapy. Typically, 10–20 passes were required to achieve complete macroscopic lesion removal. Postprocedure, 2% mupirocin ointment was applied, and patients were instructed to continue its topical use for 3 days. They were advised to keep the treated area clean and dry during this period and to adhere to strict sun protection measures afterward. Follow-up evaluations were conducted monthly until the final treatment session and again at 12-month posttreatment to assess outcomes.

**Figure 1:**
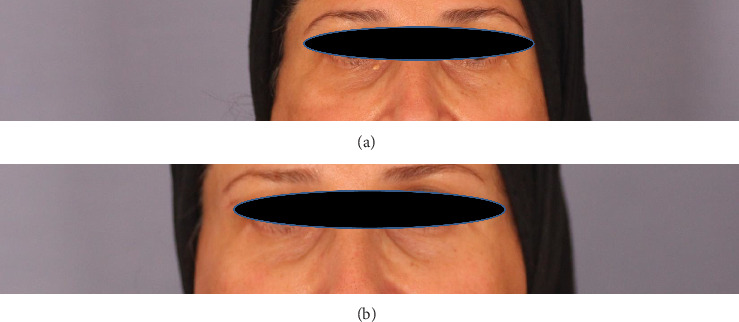
Case 1: (a) before lasering, yellow-colored papules near the inner right canthus; (b) at 12-month follow-up of the same lesion.

**Figure 2:**
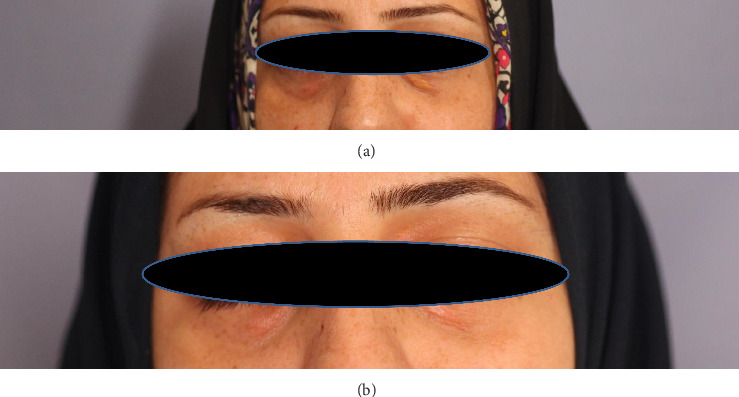
Case 2: (a) before lasering, bilateral yellow-colored plaque of the lower eyelid; (b) same lesions at 12-month follow-up.

**Figure 3:**
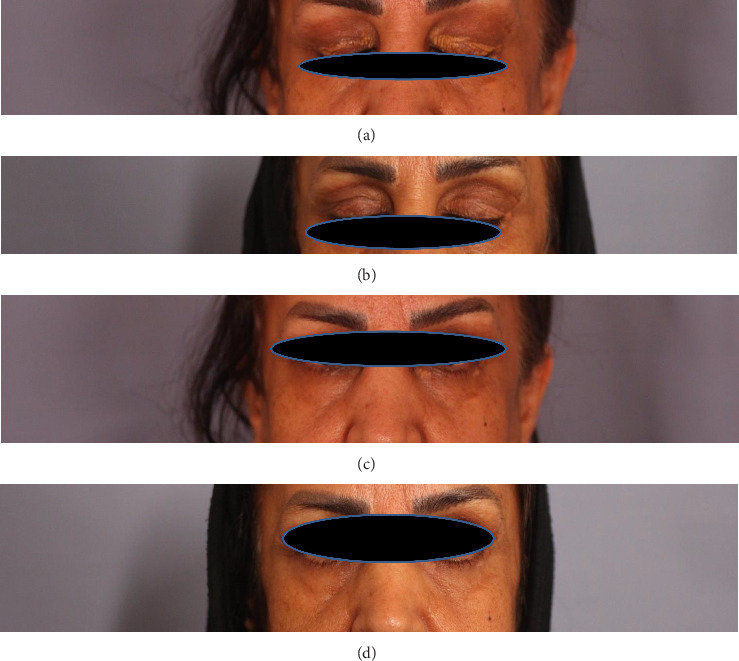
Case 3: (a) before lasering, yellow-colored papule of both upper eyelid; (b) upper eyelid at 12 months follow-up; (c) yellow-colored plaques at left inner canthus; (d) 12-month follow-up of the lower eyelid lesion.

**Figure 4:**
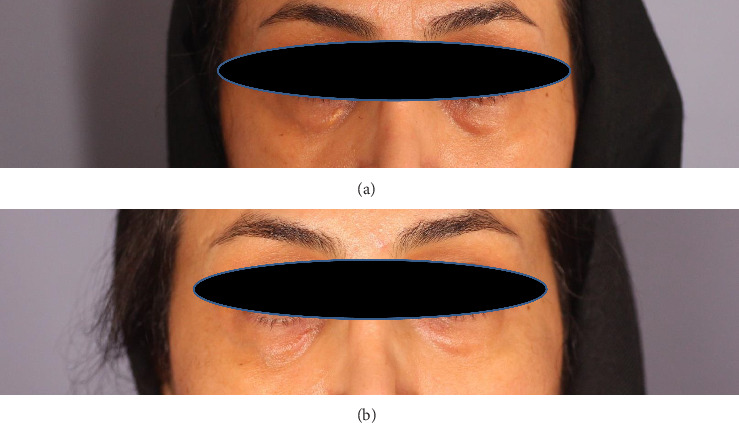
Case 4: (a) before lasering, right lower lid yellow papule; (b) at 12-month follow-up.

All lesions were cleared substantially with three sessions of Er:YAG laser treatment (Figures 1, 2, 3, and 4). None developed recurrence after 12 months of follow-up. Side effects included transient erythema and a burning sensation. None of the patients developed bleeding, scarring, or postinflammatory dyspigmentation.

## 3. Discussion

The treatment of normolipidemic and hyperlipidemic XP involves similar modalities, including surgical excision, laser therapy, electrocautery, and TCA 70% application [6]. However, these approaches often yield suboptimal outcomes, with recurrence rates as high as 40% [5]. The choice of treatment depends on the depth and extent of lipid deposition; superficial epidermal lesions may respond best to Er:YAG laser ablation, whereas lesions extending into the dermis may require CO_2_ laser therapy. Localized lesions without periorbital involvement may be more effectively managed by surgical excision [5].

Laser therapy has gained increasing preference for XP treatment due to its practicality and favorable safety profile. Various laser systems have been employed, including Er:YAG, CO_2_, Q-switched Nd:YAG, argon, and pulsed dye laser [7]. Among these, the C_2_^2^ laser has been more extensively studied than the Er:YAG laser [7]. The Er:YAG laser selectively targets epidermal lesions through tissue vaporization, with ablation continuing until pinpoint bleeding is observed. Compared to CO_2_ laser therapy, the Er:YAG laser induces a narrower coagulation zone, resulting in faster re-epithelialization, reduced postprocedural erythema, and lower risks of dyspigmentation and scarring [8].

In our study, four patients were treated with Er:YAG laser for XP. All patients reported satisfaction with the partial clearance of their lesions and experienced no recurrence at the 12-month follow-up. While our findings indicated partial lesion clearance, a more extensive study demonstrated complete clearance of 457 lesions in 214 patients with a single Er:YAG laser session [9]. In contrast to our observation of no postlaser side effects, including scarring and dyspigmentation, the larger study reported a low incidence of hypertrophic scarring (1.5%) and a recurrence rate of 9.5%. The absence of recurrence in our smaller cohort at 12 months is consistent with the low recurrence rate reported in the larger study. Notably, our study provides initial insights into patient satisfaction following Er:YAG laser treatment for XP, an aspect not extensively evaluated in prior research [9]. The difference in treatment sessions between our study (three sessions) and the larger trial (single session) reflects our conservative approach using lower fluence (7 mJ/cm^2^) tailored for Fitzpatrick skin Type IV patients to minimize risks such as postinflammatory hyperpigmentation. Our meticulous technique and postprocedure care ensured minimal complications, highlighting the importance of safety optimization in darker skin types. This underscores the need for individualized protocols based on lesion characteristics and patient demographics.

While comparative studies XP treatment modalities are still limited, Güngör et al. established comparable efficacy and safety between Er:YAG laser and TCA 70% [10]. However, a more recent study favored the Er:YAG laser over TCA 70% due to its superior efficacy and enhanced patient satisfaction, an observation consistent with the findings of our study [11]. Abdelkader and Alashry reported similar lesion clearance rates for Er:YAG and argon lasers, recommending the former for larger lesions [12]. When comparing the Er:YAG laser with the pulsed dye laser in 28 patients who were randomly assigned to both modalities, both modalities were effective at clearing the XP lesions after six sessions, two weeks apart, but the pulsed dye laser was reported to be more effective in smaller lesions [13]. The two previous comparative studies have indicated a preference for Er:YAG laser over argon and pulsed dye lasers for treating larger lesions [12, 13]. However, our studies demonstrate an acceptable clearance of even small lesions with Er:YAG laser, expanding its demonstrated utility.

Few studies compared CO_2_ and Er:YAG lasers in the treatment of XP. In a two-center randomized split-face controlled trial, Tuan et al. showed the superior efficacy of fractional CO_2_ laser over Er:YAG in the treatment of XP due to fewer treatment sessions, but the former was associated with a higher risk of complications, including hypopigmentation and scarring, compared to the latter [14]. The latter finding was compatible with the finding in our study that Er:YAG is safe, especially in darker skin types.

Re-epithelialization of the vaporized lesions needs nearly 1–2 weeks, which was seen in our study with excellent healing and without dyspigmentation or scarring.

## 4. Conclusion

Laser therapy emerged as an effective modality in treating XP because of its easy applicability and fewer overall complications. Er:YAG laser is preferred over other laser therapies as it has a faster healing time, fewer dyspigmentation, less scarring, and shorter postlaser erythema. Although the recurrence rate after Er:YAG laser for a short duration follow-up is very low, prolonged follow-up assessment is needed to assess the rate of recurrence.

## Data Availability

The data that support the findings of this study are available on request from the corresponding author. The data are not publicly available due to privacy or ethical restrictions.
